# Robot-Assisted Ileocecal Resection for Ascending Colon Cancer in a Patient With an Ileal Conduit: A Case Report

**DOI:** 10.7759/cureus.84095

**Published:** 2025-05-14

**Authors:** Junpei Takashima, Hirotoshi Kobayashi, Daisuke Fujimoto, Fumihiko Miura

**Affiliations:** 1 Surgery, Teikyo University School of Medicine Hospital Mizonokuchi, Kawasaki, JPN

**Keywords:** ascending colon cancer, ileal conduit, ileocecal resection, indocyanine green fluorescence imaging, robot-assisted surgery

## Abstract

We present a case of robot-assisted ileocecal resection for ascending colon cancer in a patient with an ileal conduit, safely performed using indocyanine green (ICG) fluorescence imaging to ensure the safe preservation of the ileal conduit. An 85-year-old woman with hematochezia was diagnosed with ascending colon cancer (cT3N0M0: stage IIA). She had undergone robot-assisted radical cystectomy with ileal conduit construction eight months earlier. Given her advanced age, a robot-assisted ileocecal resection was performed as a minimally invasive approach. Due to previous surgeries, dense adhesions around the ileal conduit and terminal ileum distorted the local anatomy, making the identification of the ileocecal region challenging. However, by modifying the surgical approach and performing meticulous dissection, the procedure was completed safely. Additionally, ICG fluorescence imaging confirmed adequate blood flow to the ileal conduit. She was discharged on postoperative day 7 without complications.

In patients with colorectal cancer who have undergone total cystectomy with ileal conduit construction, preserving the conduit's blood supply is essential, making right-sided colon cancer surgery particularly challenging. Because of these technical difficulties, open surgery is typically preferred. Nonetheless, we report the first case of robotic surgery performed in such a patient. Robotic surgery enabled the precise dissection and safe preservation of the ileal conduit, taking advantage of enhanced visualization and multi-articulated instruments. Furthermore, ICG fluorescence imaging enabled the real-time assessment of conduit perfusion, helping to avoid postoperative complications. This case highlights the feasibility of robotic surgery as a safe and effective option for colorectal cancer in patients with an ileal conduit.

## Introduction

Ileal conduit urinary diversion is the standard surgical procedure performed after total cystectomy, in which a segment of the ileum is resected from the intestinal tract while preserving its mesentery and anastomosed to the ureters, allowing urine to be diverted to a stoma on the abdominal wall [1]. However, in patients with colorectal cancer (CRC) who have undergone this procedure, right-sided colon cancer surgery is particularly challenging because preservation of the conduit's blood supply is essential, despite significant distortion of the ileocecal anatomy and dense adhesions between the ileal conduit and the surrounding ileum. Consequently, open surgery is preferred. To our knowledge, only two cases of laparoscopic surgery have been reported in such patients [2,3]. Robotic surgery for colon cancer has become increasingly widespread worldwide, and at our institution, robot-assisted surgery is the first-line treatment for CRC. However, to date, there have been no reported cases of robotic surgery for CRC in patients with ileal conduits. Herein, we present a case of ascending colon cancer in a patient with an ileal conduit that was successfully treated using robot-assisted ileocecal resection.

This case is reported in accordance with the Surgical CAse REport (SCARE) 2023 criteria [4].

## Case presentation

An 85-year-old woman presented to our hospital with a one-month history of hematochezia. Her medical history included a total abdominal hysterectomy for cervical cancer 32 years earlier and robot-assisted radical cystectomy with ileal conduit construction for bladder cancer eight months prior. Blood tests showed moderate anemia (hemoglobin 8.7 g/dL); however, carcinoembryonic antigen and carbohydrate antigen 19-9 levels were within normal range, with no other notable abnormalities. A colonoscopy revealed a type 2 lesion in the ascending colon, which was diagnosed as moderately differentiated adenocarcinoma. Contrast-enhanced chest and abdominal computed tomography (CT) showed a contrast-enhancing thickening of the ascending colon wall, without lymph node enlargement or distant metastases. The preoperative diagnosis was cT3N0M0, corresponding to stage IIA. The tumor was primarily supplied by the ileocolic artery. Considering the patient's advanced age, we judged that a minimally invasive approach would be preferable and planned a robot-assisted ileocecal resection. However, owing to the patient's history of multiple abdominal surgeries, extensive intra-abdominal adhesions were suspected. In addition, because preservation of the ileal conduit was essential, the risk of conversion to open surgery was thoroughly explained preoperatively.

**Figure 1:**
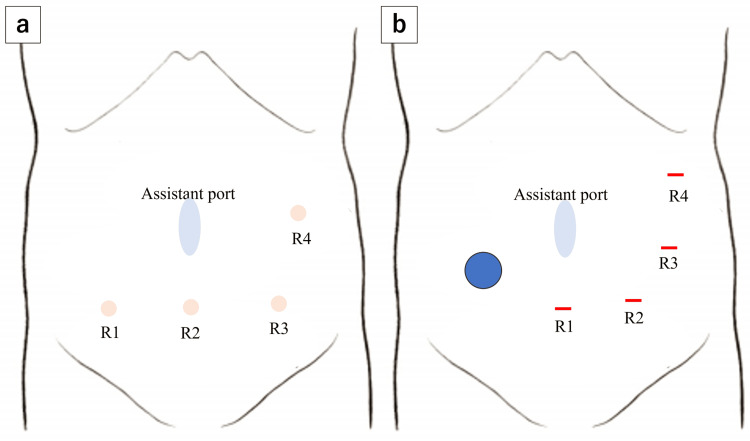
Port placement. (a) Usual port placement in our institution. A small incision is made at the umbilicus, where a 12-mm AirSeal^Ⓡ^ port (CONMED, Largo, FL, USA) is inserted and used by the assistant. Robotic ports R1-R3 are aligned transversely across the lower abdomen, with R4 placed on the left flank. (b) Port placement in the present case. Due to the presence of an ileal conduit in the right lower abdomen (blue circle), R1 was placed below the umbilicus, and the remaining robotic ports were shifted leftward accordingly. Image Credit: Authors

**Figure 2:**
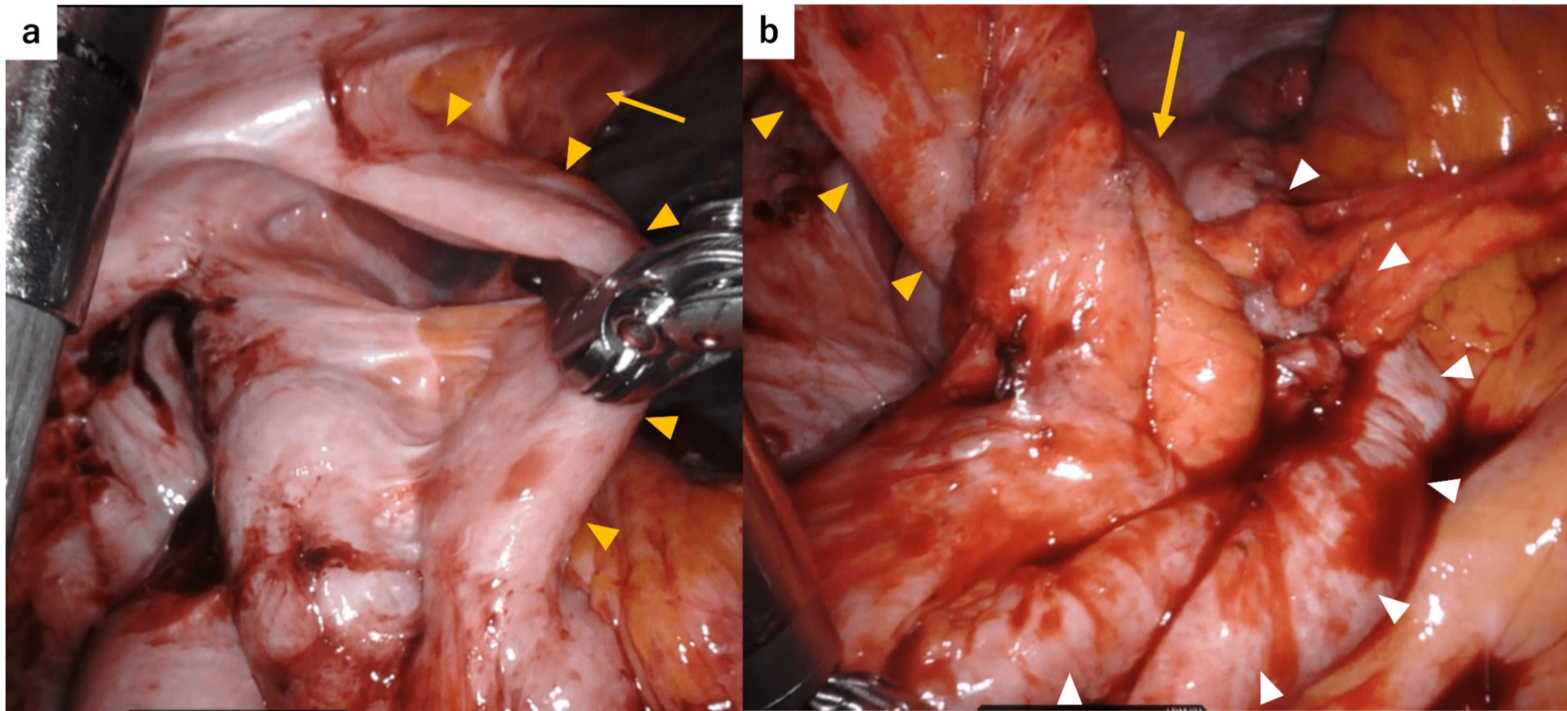
Intraoperative findings before and after the dissection and mobilization of the ileocecal region. (a) Before dissection. The anatomical boundaries between the ileal conduit (arrowheads), its mesentery (arrow), and the adjacent ileum were indistinct. The course from the terminal ileum to the cecum could not be clearly identified. (b) After dissection. The ileal conduit and its mesentery (arrowheads) became clearly visible, with the adjacent terminal ileum surrounding them (white arrowheads). The cecum and ascending colon (arrow) were identified lateral to the ileal conduit.

**Figure 3:**
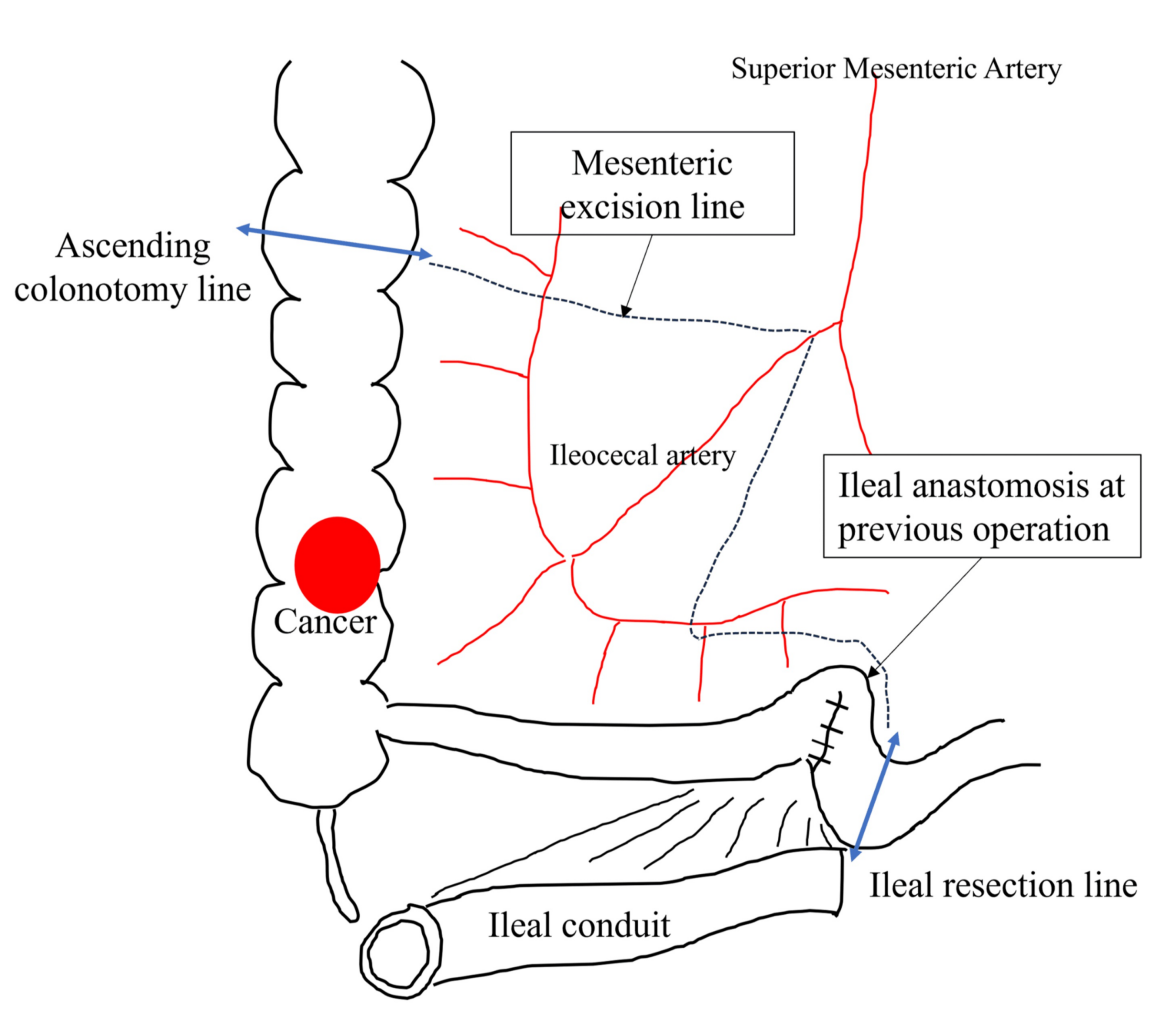
Resection line of the bowel and mesentery. The bowel was resected from the ascending colon, 10 cm distal to the tumor, to the ileum just proximal to the previous anastomosis created during ileal conduit construction (double-headed arrows). The mesentery was also resected accordingly (dotted lines). Image Credit: Authors

**Figure 4:**
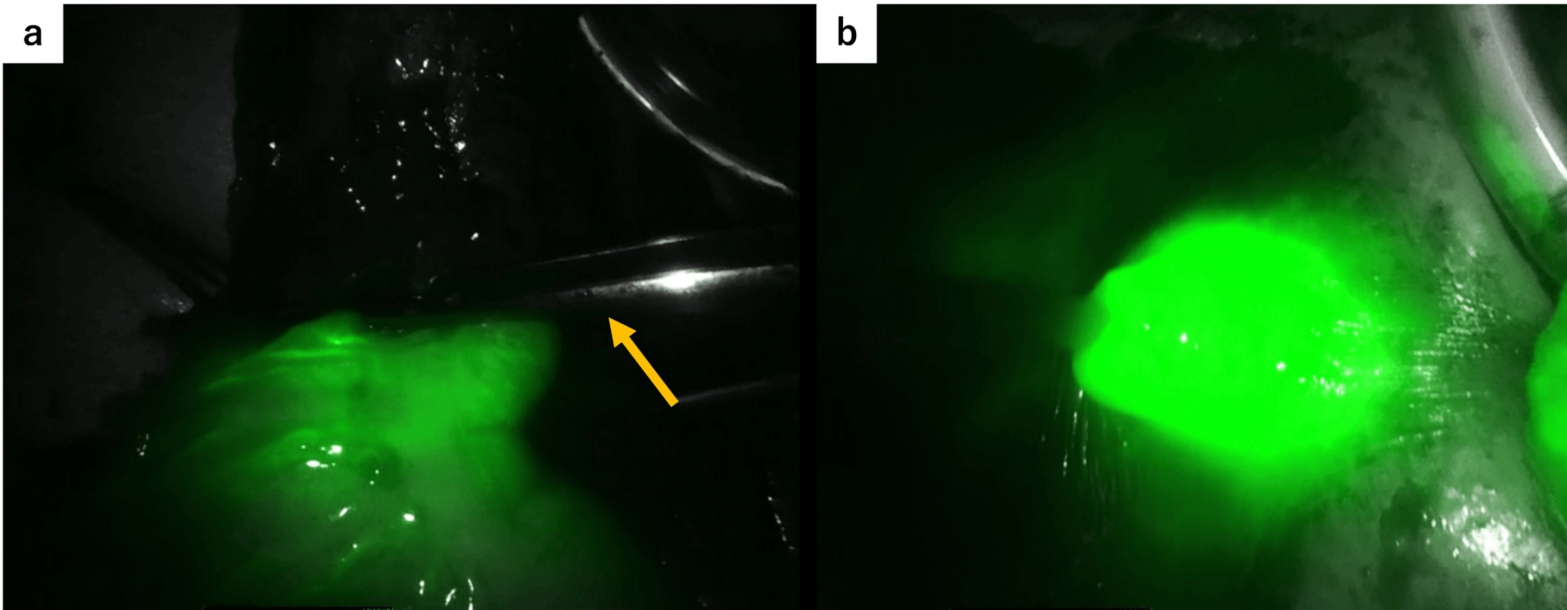
Intraoperative assessment of bowel perfusion using ICG fluorescence imaging. (a) The planned transection line of the ascending colon is indicated by forceps (arrow). Fluorescence was observed at this site, confirming adequate perfusion. (b) Fluorescence signal was also detected in the ileal conduit, indicating preserved blood flow. ICG: indocyanine green

Robot-assisted ileocecal resection was performed using the da Vinci Xi system (Intuitive Surgical, Sunnyvale, CA, USA). Owing to the presence of the ileal conduit in the lower right abdomen, the ports were placed more toward the left side than usual, as shown in Figure 1a, 1b. Extensive adhesions were observed between the greater omentum and the abdominal wall, and we prioritized their dissection at the beginning of the procedure. Our department routinely adopts the retroperitoneal approach for ileocecal resection. In the head-down position, we attempted to shift the small intestine cranially to clear the surgical field, but adhesions were found between the small intestine and the deep pelvic wall, which were carefully dissected. To avoid interference with the ileal conduit, retroperitoneal dissection was initiated near the duodenum, away from the conduit, and progressed in the cranial and lateral directions. We then proceeded to mobilize the ileocecal region, where we encountered dense adhesions between the mesentery of the ileal conduit and that of the terminal ileum, making the identification of the individual mesenteries extremely difficult, as shown in Figure 2a. According to the operative records from her previous bladder cancer surgery, the ileal conduit had been constructed using the distal ileum, approximately 20-35 cm from the ileocecal valve. Dissection between the ileal conduit and the adjacent small intestine was performed with great care to avoid injury to the mesentery of the conduit. This allowed us to clearly identify the anatomy of the ileocecal region, including the uretero-conduit anastomosis, as shown in Figure 2b. We then shifted the operative field from the retroperitoneal approach to a medial approach, divided the ileocolic artery at the right border of the superior mesenteric vein, and performed a D2 lymphadenectomy. After the mobilization of the hepatic flexure, we proceeded to extracorporeal manipulation. We planned to resect the bowel from the ascending colon 10 cm distal to the tumor to the ileum just proximal to the previous anastomosis created during ileal conduit construction, as shown in Figure 3. Following mesenteric dissection, 5 mL of indocyanine green (ICG) was intravenously administered. Using a Firefly imaging system, we evaluated the perfusion of both the bowel segments scheduled for anastomosis and the ileal conduit. Fluorescence was observed within 20 seconds, confirming adequate blood flow, as shown in Figure 4a, 4b. The bowel was then resected and anastomosed. The total operative time was four hours and 40 minutes with minimal blood loss.

The patient's postoperative course was uneventful, and she was discharged on the postoperative day 7. Postoperative pathological findings showed moderately > well > poorly differentiated adenocarcinoma with subserosal invasion and no lymph node metastasis (pT3N0M0), along with negative circumferential and longitudinal margins (R0). No adjuvant chemotherapy was administered, and she has remained recurrence-free for 15 months after surgery.

## Discussion

To our knowledge, there have been no previous reports of robot-assisted surgery for CRC in patients with an ileal conduit. We successfully performed a robot-assisted ileocecal resection for ascending colon cancer in such a patient, using ICG fluorescence imaging to ensure the safe preservation of the ileal conduit.

Recently, robot-assisted surgery for colon cancer has become increasingly widespread worldwide. In Japan, it has been covered by the National Health Insurance since April 2022, and its use continues to expand. Although robotic surgery tends to require longer operative times compared to laparoscopic surgery, several studies have suggested its advantages in terms of lower conversion rates and reduced postoperative complications, indicating favorable short-term outcomes [5]. At our institution, robotic surgery is the first-line treatment for CRC. In this case, we selected robotic surgery as a minimally invasive option, given the patient's advanced age and a history of decreased activities of daily living (ADL) following a previous radical cystectomy.

In performing this surgery safely, we identified several technically important considerations. First, the port placement was crucial. To avoid interference with the ileal conduit, the ports were positioned further toward the left side than usual (Figure 1a, 1b). Although the robotic arms were slightly closer than normal, requiring caution to avoid arm collisions, there was no interference with the ileal conduit, and the procedure was completed safely. Second, accurate anatomical recognition was essential. In this case, previous surgeries had significantly distorted the anatomy of the ileocecal region, necessitating more careful and extensive dissection than usual. We first dissected the dense adhesions involving the omentum, small intestine, pelvic wall, and abdominal wall. During the retroperitoneal approach, dissection was initially performed around the duodenum, postponing the mobilization of the ileocecal region until later. This sequence allowed for a clearer identification of the surrounding anatomical structures. Third, preservation of the mesentery and blood flow to the ileal conduit was critical. In this case, dense adhesions were found between the ileal conduit and the surrounding ileum (Figure 2a), and we paid close attention to the careful dissection between these structures to avoid compromising the conduit's vascular supply.

Given these technical considerations, open surgery is generally preferred for ascending colon cancer in patients with an ileal conduit. Although several reports have addressed laparoscopic surgery in patients with CRC who had had previously undergone abdominal surgery [6,7], very few have examined cases involving an ileal conduit. To the best of our knowledge, only two cases of laparoscopic surgery for CRC have been reported in such patients [2,3], and this is the first report of robot-assisted surgery in this patient group. In previously reported laparoscopic cases by Okoshi et al. [2] and Suzuki et al. [3], operative times were 292 and 308 minutes, with blood loss of 152 mL and 70 mL, respectively. No complications occurred in either case, and the postoperative hospital stay was 12 days in the latter case, while not reported in the former. In contrast, an open case by Okada et al. required 367 minutes with 520 mL of blood loss and mild postoperative paralytic ileus, resulting in discharge on postoperative day 15 [8]. Our robotic approach achieved a shorter operative time, minimal blood loss, and an earlier discharge without complications. While direct comparisons are limited by the nature of case reports, these findings suggest the potential advantages of robotic surgery in managing technically demanding cases in patients with an ileal conduit.

In the present case, several features of the robotic system contributed to the safe and effective performance of the procedure. Robotic systems offer multi-articulated instruments, high-definition 3D magnified visualization, tremor filtration, and motion scaling, allowing for more precise procedures than conventional laparoscopy. In particular, the multi-articulated functionality of robotic instruments was effective during dissection between the ileal conduit and surrounding small intestine. Although laparoscopic dissection is limited to a unidirectional approach, robotic surgery allows for multidirectional manipulation, which is advantageous for managing the complex adhesions encountered in this case. Furthermore, the da Vinci Xi system is equipped with a Firefly imaging system, enabling the real-time visualization of blood flow. This feature was especially useful in confirming the preservation of vascular supply to the ileal conduit. The patient experienced no postoperative complications related to the conduit.

One of the disadvantages of robotic surgery is the lack of tactile feedback. This limitation raises concerns about the potential for inadvertent organ injury due to excessive force during tissue manipulation [9]. Surgeons must be particularly mindful of this risk, and conversion to open surgery should not be hesitated if intraoperative findings warrant it. However, in the present case, robotic surgery was safely performed by taking full advantage of its strengths, even in a patient with CRC and an ileal conduit. The patient was discharged uneventfully on postoperative day 7 without any decline in ADL, suggesting that the minimally invasive nature of robotic surgery contributed to the favorable outcome.

## Conclusions

Robot-assisted ileocecal resection may be a safe and feasible option even for ascending colon cancer in patients with an ileal conduit. Although surgery in patients with an ileal conduit is technically challenging due to distorted anatomical structures and the need to preserve blood flow to the conduit, in this case, the surgery was successfully completed without complications by utilizing the features of robotic surgery and implementing meticulous intraoperative strategies.
